# Factors That Influence Access to Medical Assistance in Dying Services: An Integrative Review

**DOI:** 10.1111/hex.70058

**Published:** 2024-10-17

**Authors:** Jayne Hewitt, Michael Wilson, Ann Bonner, Melissa J. Bloomer

**Affiliations:** ^1^ School of Nursing and Midwifery Griffith University Southport Queensland Australia; ^2^ Queen Elizabeth II Hospital, Metro South Health and Hospital Service Coopers Plains Queensland Australia; ^3^ Law Futures Centre Griffith University Nathan Queensland Australia; ^4^ University of Adelaide Nursing School Adelaide Australia; ^5^ Kidney Health Service, Metro North Hospital and Health Service Herston Queensland Australia; ^6^ Intensive Care Unit Princess Alexandra Hospital, Metro South Health and Hospital Service Woolloongabba Queensland Australia

**Keywords:** assisted dying, euthanasia, health service access, integrative review, MAiD, supply and demand

## Abstract

**Background:**

In nearly all jurisdictions where it is permitted, Medical Assistance in Dying is situated in a healthcare system. Currently, limited evidence demonstrates how supply and demand factors influence access to Medical Assistance in Dying.

**Objective:**

The aim of this study is to synthesise empirical research from jurisdictions where Medical Assistance in Dying is legal to identify how supply and demand factors influence access for eligible adults.

**Method:**

An integrative review was conducted. CINAHL Complete, PubMed, ProQuest, PsycINFO and Embase databases were systematically searched for studies published between January 1998 and January 2024. Records were independently assessed against inclusion and exclusion criteria. Additional studies were identified by forward and backward citation searching. All studies were assessed for quality. Findings were analysed deductively using an established conceptual framework, and a secondary narrative synthesis was undertaken.

**Results:**

Fifty‐eight studies met the inclusion criteria. Most studies (*n* = 32) reported results related to the supply side, 16 reported on the demand side and 10 reported on both supply and demand dimensions of access. Studies about supply showed that health service policies may obstruct access to Medical Assistance in Dying. For healthcare professionals, the practice entails an additional workload and can create tensions with colleagues. Studies of the demand for Medical Assistance in Dying focused on supporting time‐critical decisions, adequate planning and caregiver support.

**Conclusion:**

Access to Medical Assistance in Dying requires the participation of health services and healthcare professionals but is hindered by policies that obstruct access and direct financial and indirect emotional labour costs. Innovative and inclusive models to promote high‐quality, compassionate care at the end of life and access to Medical Assistance in Dying should be considered.

**Patient or Public Contribution:**

Patients, caregivers and service users were involved in many of the studies included in this review, and their experiences and perspectives contributed to the analysis and synthesis in this review.

## 1 Background

Medical Assistance in Dying (MAiD) has become an established legal choice for eligible adults in more than 25 jurisdictions across 12 countries [1]. MAiD is known by various legal and cultural terms such as euthanasia, voluntary assisted dying or physician‐assisted dying [2], but the acronym MAiD, which evolved in North America, is used in this article. The practice involves intentionally ending the life of a person at their request by the administration of a prescribed substance that is self‐administered or administered by healthcare professionals (HCPs) [2]. Laws permitting MAiD are underpinned by the principle of respect for individual autonomy, the temporality of natural death and the experience of suffering [3]. To be eligible to access MAiD, a person suffering at the end of life must meet specified legal criteria and complete the process required by law [4]. Medical practitioners, and in Canada and one Australian Territory, nurse practitioners, are authorised to assess whether a person requesting MAiD meets the criteria and are responsible for ensuring the procedural elements of the process are followed [4].

Consequently, although the provision of MAiD is qualitatively different from other healthcare practices as it does not seek to cure illness or suffering, it is generally situated in healthcare [3]. Access to this service results from a person's perceived need for MAiD and the opportunity to reach and use the service. Characteristics of the person, their social and physical environments, health systems and the support of providers will also influence access [5]. In the case of MAiD, how laws that govern the process are framed is also a consideration [3].

With the increasing number of jurisdictions that permit MAiD, there has been a corresponding increase in research about MAiD. Acknowledging that support and participation of HCPs are central to MAiD access, particularly those authorised to assess eligibility, recent reviews have reported on the attitudes of medical practitioners to MAiD [6], the experiences of [7] and the impacts on HCPs who participate in MAiD [3]. Concern for the emotional and moral well‐being of HCPs involved in MAiD has been a consistent finding in these reviews [3, 7].

In those jurisdictions where MAiD is relatively new, other literature reviews report on the barriers and facilitators to implementing the service in clinical practice [8, 9]. These reviews identified barriers to implementation, such as the burdens associated with complying with legal ‘safeguards’, including assessing whether the request for MAiD is coerced [9], but also highlighted how clear and specific guidance to support HCPs enabled availability. Although the concept of access may have been implicit in each review, the factors influencing access were not explicitly identified. The aim of this review was to synthesise empirical research from jurisdictions where MAiD is legal to describe the factors that influence access to MAiD for eligible adults. The research question guiding the study was as follows: what are the supply‐ and demand‐side factors that influence access to MAiD for eligible adults?

## 2 Methods

**Figure 1:**
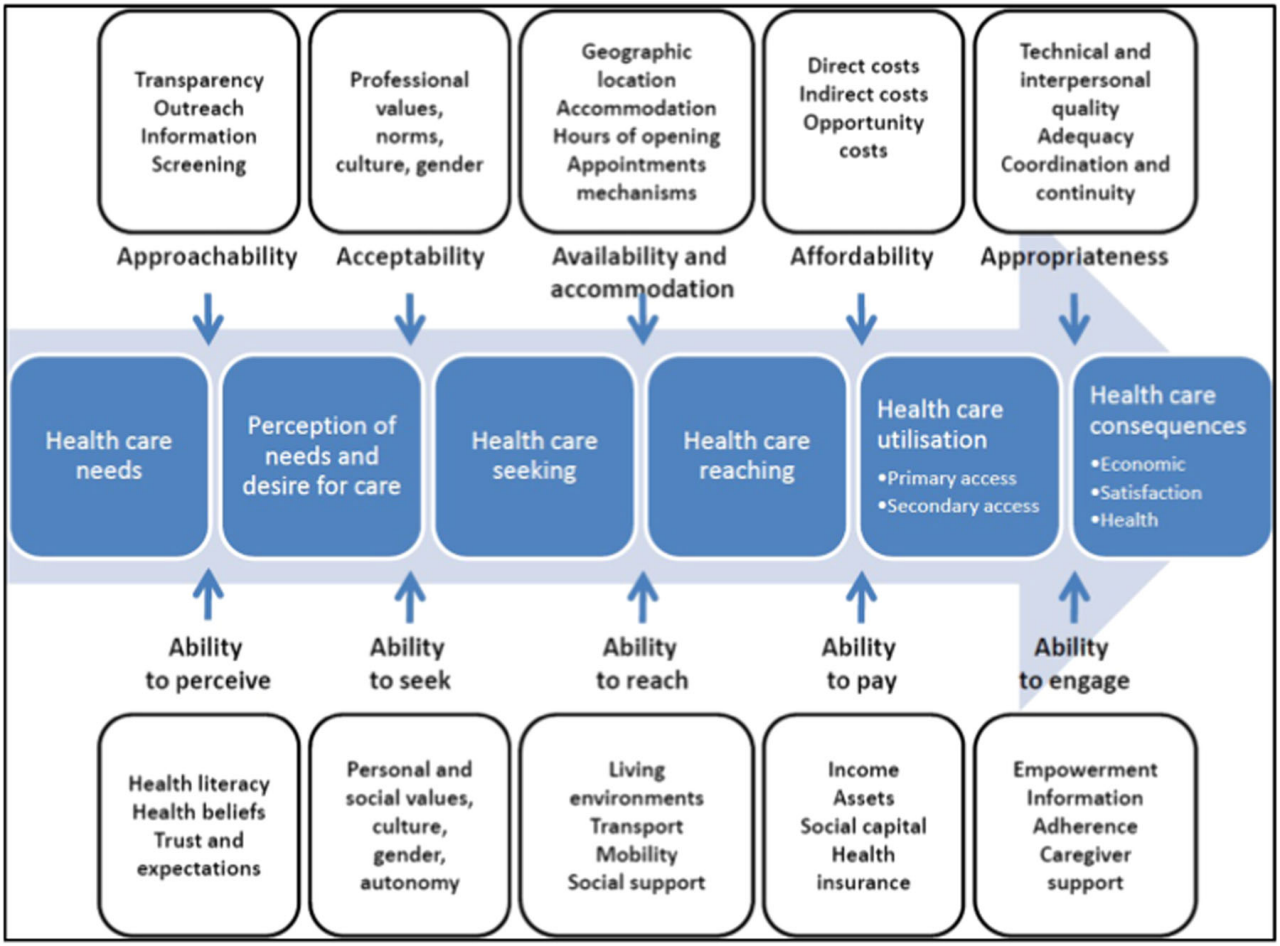
Healthcare Access Framework. Reprinted from Levesque et al. [5] with permission from BioMed Central Ltd (copyright licensee).

Levesque et al. [5] conceptualised access as the opportunity to identify, seek, reach and use healthcare services, and developed literature‐informed healthcare access framework based on dimensions and abilities that operationalise a spectrum of access to healthcare (see Figure 1). In the framework, the supply‐side dimensions reflect what is expected of those providing healthcare services and the abilities of those seeking access reflect demand‐side factors.

This framework has been used in studies of various healthcare services, such as primary care [10], care for people with chronic conditions [11] and the geographical impacts of access to specialty healthcare [12]. It has also recently been used to explore the potential impact of conscientious objection by healthcare institutions on access to MAiD [13]. Building on this work, the healthcare access framework is used to identify the factors affecting access to MAiD. This review is reported using the Preferred Reporting Items for Systematic Reviews and Meta‐Analyses (PRISMA) statement [14], and the review protocol was prospectively registered with Prospero (CRD42022382185). Publications that reported primary research about using or participating in MAiD in jurisdictions where the practice is authorised by legislation, involving adult participants who were patients, family members or proxy decision‐makers, or HCPs of any discipline, including non‐clinical leaders, published in English between January 1998, 2 months after the first assisted dying law was enacted in the US state of Oregon [15], and January 2024 were eligible for inclusion.

Search terms were developed with the guidance of a specialist health librarian and included ‘euthanasia, active, voluntary’ OR ‘suicide, assisted’ AND ‘health services accessibility’ OR ‘delivery of healthcare’. Medical subject headings (MeSH) terms were used where available. The electronic databases CINAHL Complete, PubMed, ProQuest, PsycINFO and Embase were searched, and then forward and backward citation searching was undertaken. An example search string is included as a File S1. Citations were deduplicated first in EndNote (V9) [16] and then transferred to Covidence [17], where two authors (J.H. and M.W.) independently screened the titles and abstracts against the inclusion criteria. Full texts of the remaining citations were accessed, read and screened. Conflicts were resolved through discussion with a third author (M.J.B.).

All included studies were independently evaluated using the Caldwell et al. quality appraisal framework, which uses 11 criteria to assess the strengths and weaknesses of each study [18]. Two authors (J.H. and M.W.) scored each study independently. Data were extracted from the studies into a descriptive charting matrix using Microsoft Excel to summarise study characteristics and the supply‐ and demand‐side dimensions of the Healthcare Access Framework that were addressed [5]. Two authors (J.H. and M.W.) independently checked the extracted data for accuracy and consistency. Results were discussed by three authors (J.H., M.W. and M.J.B.) in terms of major findings within each existing dimension. To deductively analyse and then synthesise the data, quantitative findings were converted into qualitative form and then combined with the qualitative findings [19].

## 3 Results

**Figure 2:**
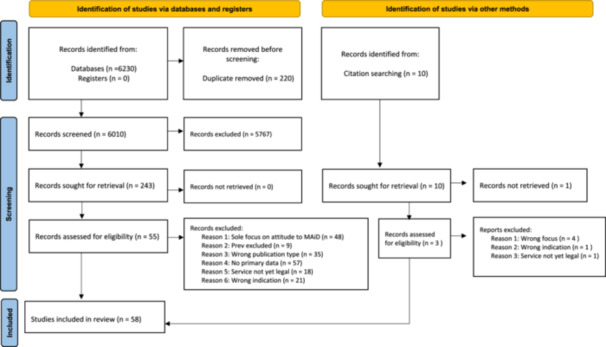
PRISMA flow diagram. From: Page et al. [14].

Fifty‐eight studies were included in the review. Six studies analysed textual data from legislation and policy documents. 52 studies collected participant data, and collectively reported the views of practices of 8503 stakeholders (median per study = 45) regarding access to MAiD (see Figure 2—PRIMSA flow diagram). Data from patients and/or families were included in 35% (*n* = 18) of the studies, solely medical practitioners in 29% (*n* = 15), a range of HCPs in 17% (*n* = 9), HCPs and other stakeholders in 15% (*n* = 8) and institutional providers were used 4% (*n* = 2) of the studies.

Studies were conducted in Canada (*n* = 22) [20, 21, 22, 23, 24, 25, 26, 27, 28, 29, 30, 31, 32, 33, 34, 35, 36, 37, 38, 39, 40, 41]; the United States of America (*n* = 12) [15, 42, 43, 44, 45, 46, 47, 48, 49, 50, 51, 52]; The Netherlands (*n* = 11) [53, 54, 55, 56, 57, 58, 59, 60, 61, 62, 63]; Australia (*n* = 7) [64, 65, 66, 67, 68, 69, 70]; Belgium (*n* = 2) [71, 72]; Switzerland (*n* = 2) [73, 74] and New Zealand (*n* = 1) [75]. One international study reported on data collected in Europe, North America and Australia [76]. Studies were conducted between 2004 and 2023, with more than half (*n* = 30) between 2020 and 2023. Fifty percent (*n* = 29) of studies used qualitative methods [15, 21, 22, 23, 28, 29, 33, 36, 38, 41, 42, 43, 48, 50, 51, 55, 57, 58, 59, 62, 64, 65, 66, 67, 68, 69, 70, 74, 75], 35% (*n* = 20) used quantitative methods [20, 30, 31, 32, 34, 35, 39, 40, 44, 47, 49, 52, 53, 54, 56, 60, 61, 63, 71, 73] and 14% (*n* = 9) used a mixed‐methods approach [24, 25, 26, 27, 37, 45, 46, 72, 76]. Most qualitative studies (69%, *n* = 20) focused on supply factors, whereas quantitative studies (45%, *n* = 9) generally focused on demand factors. Just over half (55%, *n* = 5) of the mixed‐method studies focused exclusively on supply factors, and the rest explored both supply and demand factors, but none focused solely on demand factors. Study characteristics are included in Table S1. The mean appraisal of study quality score was 9.4 (out of 11), with mixed‐method designs scoring the highest. A decision was taken a priori not to exclude a study based on quality assessment but to consider the appraisal in the synthesis. The quality appraisal scores are included in Table S2.

### 3.1 Distributions of Studies Across the Framework

Of these 58 studies, 55% (*n* = 32) reported results related to the supply‐side dimensions, 28% (*n* = 16) reported on demand‐side dimensions and 17% (*n* = 10) reported on issues relating to both. Chart reviews or retrospective data studies were often used to examine the experiences of requesting patients or families [30, 54]. Table 1 shows how supply and demand dimensions were addressed in each study. Representative findings that highlight the meaning of each dimension are included in Table S3.

**Table 1:** Supply‐ and demand‐side dimensions assessed.

	Supply‐side dimensions					Demand‐side dimensions					
Reference, country	Approachability	Acceptability	Availability and Accommodation	Affordability	Appropriateness	Ability to perceive	Ability to seek	Ability to reach	Ability to pay	Ability to engage	Total
Antonacci et al. [20], Canada	✓				✓						2
Back et al. [42], United States						✓	✓			✓	3
Beernaert et al. [71], Belgium						✓	✓			✓	3
Bergman et al. [53], The Netherlands	✓	✓			✓						3
Boivin et al. [21], Canada	✓	✓			✓		✓			✓	5
Bolt et al. [54], The Netherlands						✓	✓	✓			3
Borgsteede et al. [55], The Netherlands	✓				✓	✓	✓				4
Bouthillier and Opatrny [22], Canada	✓	✓									2
Brown et al. [23], Canada	✓						✓	✓		✓	4
Brown et al. [24], Canada		✓	✓	✓	✓						4
Brown, et al. [25], Canada	✓	✓		✓	✓						4
Buchbinder et al. [43], United States							✓	✓		✓	3
Buiting et al. [56], The Netherlands					✓						1
Cain et al. [44], United States	✓										1
Campbell and Black [45], United States	✓	✓									2
Campbell and Cox [46], United States	✓										1
Campbell et al. [47], United States	✓	✓	✓		✓						4
Close et al. [64], Australia		✓	✓	✓							3
De Boer et al. [57], The Netherlands		✓	✓	✓	✓						4
Dees et al. [58], The Netherlands	✓	✓			✓	✓				✓	5
Dion, et al. [26], Canada		✓	✓		✓			✓		✓	5
Dobscha et al. [48], United States	✓	✓	✓	✓	✓						5
Fischer et al. [73], Switzerland	✓					✓				✓	3
Frolic et al. [27], Canada		✓			✓			✓		✓	4
Gamondi et al. [74], Switzerland	✓	✓	✓		✓						4
Ganzini et al. [49], United States						✓	✓				2
Gerson et al. [50], United States	✓	✓						✓	✓		4
Haining et al. [65], Australia	✓	✓	✓	✓							4
Khoshnood et al. [28], Canada		✓		✓							2
Kortes‐Miller and Durant [29], Canada	✓	✓	✓	✓	✓						5
Kusmaul, et al. [15], United States	✓										1
Lees et al. [31], Canada							✓			✓	2
Lees, et al. [30], Canada						✓		✓			2
Lemiengre et al. [72], Belgium	✓	✓									2
Munro et al. [32], Canada						✓	✓				2
Oczkowski et al. [33], Canada	✓	✓									2
Oliver et al. [76], International	✓	✓	✓		✓						4
Pearlman et al. [51], United States						✓	✓				2
Perron et al. [34], Canada	✓	✓	✓		✓						4
Redelmeier et al. [35], Canada									✓		1
Roest and Leget [59], The Netherlands	✓	✓		✓	✓						4
Ruijs et al. [60], The Netherlands						✓					1
Rutherford et al. [66], Australia	✓	✓	✓		✓						4
Sellars et al. [67], Australia	✓	✓	✓	✓	✓						5
Shaw et al. [36], Canada	✓	✓	✓	✓	✓						5
Silvius, et al. [37], Canada	✓	✓	✓								3
Smith et al. [52], United States						✓	✓			✓	3
Snelling et al. [75], New Zealand		✓	✓	✓	✓						4
Snijdewind et al. [61], The Netherlands			✓					✓			2
ten Cate et al. [62], The Netherlands	✓				✓						2
Thomas et al. [38], Canada	✓	✓									2
Trachtenberg and Manns [39], Canada									✓	✓	2
Tran et al. [40], Canada									✓		1
van den Ende et al. [63], The Netherlands						✓				✓	2
White et al. [68], Australia	✓				✓						2
White et al. [69], Australia				✓			✓	✓	✓		4
White et al. [70], Australia						✓	✓	✓			3
Wiebe et al. [41], Canada	✓	✓	✓		✓						4

### 3.2 Supply‐Side Dimensions Influencing Access to MAiD

The supply dimension, *approachability*, indicates whether a health service is transparent about offering MAiD. The extent to which a chosen stance was publicly known was variable. Although some studies reported that providers' policies were often silent on this issue [45, 72], there was an expectation from service users that institutions make their position on MAiD publicly known [38, 64]. Where information was publicly known, people could make informed decisions about options for care at the end of life [38]. In some jurisdictions, system‐level supports such as communication tools and standardised care pathways have been implemented to address these approachability barriers [25, 33]. Services that assist patients, families and providers in navigating MAiD processes, including locating a willing provider, are common features of Australian and Canadian health services where MAiD is comparatively recent [37, 67].

*Acceptability* relates to social norms or beliefs about aspects of the service [5]. For instance, medical practitioners may agree with MAiD in principle, but this does not necessarily mean that they are willing to participate in all MAiD‐related tasks [47]. Factors influencing their willingness to participate included patient characteristics such as accumulation of age‐related problems, including dementia, certain diagnoses like heart failure or the dominance of existential issues such as being ‘tired of living' [66]. Time and workload requirements, lack of remuneration and perceived reputational risks also influenced their willingness to participate [24, 29]. Studies reported that medical practitioners who participated in MAiD found it personally or professionally fulfilling [47] despite the challenge that it presented to usual medical practice [66]. However, even if HCPs are prepared to participate in MAiD, their ability to do so may be hindered by non‐government providers, usually faith‐based organisations, that restrict participation in MAiD because it conflicts with the values and beliefs underpinning the institution [44, 46]. This conflict then negatively impacts service users (patients) who seek MAiD [70].

Facilities and services that can be reached in a timely manner and the characteristics of providers refer to the *availability* of a healthcare service [5]. Availability was influenced by expansive geographies [26] and/or those affected by the COVID‐19 pandemic [41, 76]. The timely delivery of MAiD can be impacted by procedural requirements and the need for rigorous eligibility assessments [69, 75]. The time to develop trusting relationships [57], provision of end‐of‐life care resources and support for participating HCPs [25] also affected availability. A medical practitioner participant in one study commented, ‘Yeah, there is a huge gap between idealistic agreement with a thought and being involved in it. It's just an immeasurable gap that I hadn't anticipated’ [48, p. 454].

*Affordability* relates to the direct and indirect costs of participating in MAiD. For HCPs, direct costs related to remuneration did not match the time commitment required to provide this service [28, 36]. One medical practitioner described the impact of time costs:

…the short stay admission probably took me 16 hours to arrange because then I had to do in‐services with the staff, where it's going to occur, talk to the director of admissions, try and arrange a bed, verify the bed, confirm the bed, and then deal with pharmacy and how we were going to [do] this because [she was] not a patient in the system because she's an outpatient. [36, p. e397]

Indirect costs often reflected the impacts of emotional labour and included counterpressure from relatives and employers [57]. The intensity of decision‐making [48, 50] and threats to practitioners' well‐being and collegial relationships [28, 67] were other indirect costs.

The final supply‐side dimension, *appropriateness*, denotes the alignment of services with the person's needs [5]. Although a person suffering at the end of life may seek MAiD, whether this is appropriate is determined by eligibility criteria set out in law. Studies among Dutch medical practitioners identified significant variability in abilities to assess eligibility [56], particularly in interpreting legal criteria, such as what constitutes unbearable suffering [62]. Soon after Oregon's Death with Dignity law was implemented, medical practitioners found themselves unprepared for managing requests and helping with symptom control while remaining committed to supporting the patient. However, these deficiencies were mitigated as experience with MAiD increased [48].

### 3.3 Demand‐Side Dimensions Influencing Access to MAiD

A person's *ability to perceive a need* for the service is affected by their health literacy, health beliefs and knowledge of healthcare systems [5]. Most studies that addressed the ability to perceive a need for MAiD focused on exploring reasons for seeking it, the situations of those requesting it and the strength or durability of the desire [30, 32]. A perceived need for MAiD was due to a fear of increasing pain, dependency, loss of sense of self or a need to control the manner of death [42, 43, 51]. Pearlman et al. report one patient participant's perceived need for MAiD, ‘[T]here's no question about wanting to make provisions for a hastened death should conditions become so unbearable. I want to spare my family as much of that grief as I can’ [51, p. 238]. The clarity of the perceived need for MAiD contributed to successful access. Studies among Dutch patients found that once a request for MAiD had been formalised, it tended to persist through to completion [63], with a significant completion rate in cases where the request was present in an advance care directive [54].

*The ability to seek* relates to personal autonomy and the capacity to weigh this option among others [5]. For some, the potential loss of autonomy contributes to their experience of unbearable suffering and preference for MAiD [49]. Having decided to access MAiD, the desire can persist even if an initial request is denied [63]. However, if the request is made late in the disease trajectory, the odds of completing the process are significantly reduced [30]. A decision may be made autonomously, although people seeking access to MAiD may require emotional and instrumental support from caregivers [43], particularly when an institutional provider objects to MAiD that could require end‐of‐life care to be provided at home [70].

Physical mobility, schedule flexibility and knowledge of who can provide the service affect a person's *ability to reach* [5]. When a person's mobility is severely restricted, this can be a motivator for seeking MAiD and an obstacle to reaching it [73]. Two studies reported on the ability to reach the service by highlighting the benefits of telemedicine, where geographic realities made travel impossible [26], and overcoming the isolation constraints of the COVID‐19 pandemic [41].

*The ability to pay* reflects the patient's capacity to meet the costs associated with the service [5]. Gerson et al. [50] reported that when insurers would not cover the high cost of the medication, patients incurred significant out‐of‐pocket expenses, which had the perverse effect of promoting suicide among hospice patients. Research from Canada, where the service is publicly funded, is equivocal about the impact of a person's financial status on access to MAiD [40]. In one Canadian health service catchment, most requests for MAiD came from people in lower socioeconomic groups, but financial status did not affect the proportion of people who ultimately received MAiD [40]. In contrast, others report that MAiD was proportionately less frequent for those with low socioeconomic status [35].

*The ability to engage* refers to the participation and involvement of the person in decision‐making and includes notions of health literacy [5]. This dimension shared shades of meaning across the framework; for example, some studies that considered this dimension also focused on the ability to perceive the need for MAiD [52, 58, 71]. The ability to engage was contingent on developing and maintaining a respectful therapeutic relationship with a HCP so that options for MAiD, including limits to participation, could be openly discussed [42]. The desire to discuss MAiD was higher when the physical symptoms of the illness were unbearable [71]. Engagement frequently requires the active participation of caregivers even if they feel unprepared for the role [43]. Including family and caregivers in discussions helped them to provide the emotional and instrumental support that a person seeking MAiD requires [43].

## 4 Discussion

Although MAiD is a legal end‐of‐life option in many jurisdictions, taking steps to end a person's life at the request of patients challenges traditional goals of healthcare [66] and creates unique issues of access. Using Levesque et al's Healthcare Access Framework [5] enabled consideration of the supply‐ and demand‐side factors that influence access to MAiD. Supply access factors were the extent to which health services offer MAiD and HCPs decisions about their participation; from the demand side, patients' health literacy and ensuring support to access MAiD was available where necessary were common factors.

Health services restricting access to MAiD (supply) arose from explicit prohibitions due to institutional objection [44, 50] or through policy‐based decisions, such as requiring palliative care or mental health consultations, which are framed as ‘safeguards’ [44]. Although HCPs may choose not to participate in MAiD because of a conscientious objection, whether health services can also refuse to provide MAiD on this basis is contested [77]. It has been argued that a health service's right of self‐governance (rather than conscience) allows discretion over the services that it will and will not provide [78]. Other reasons for refusing to provide MAiD included a lack of resources or capacity or that MAiD conflicts with a health service's belief that palliative (hospice) care intends neither to hasten nor postpone death [46]. Given the profound impact that a health service decision to avoid MAiD can have [70], it is incumbent on health services to be transparent about whether MAiD is possible in that location and consider implementing strategies to address access barriers.

Over time, the effect of health services' decisions to refuse to offer MAiD has become apparent. For example, in Canada (British Columbia), standalone hospice centres in remote areas are owned by large faith‐based corporations, and because these hospices can refuse to offer MAiD, dying people must travel to other regions for assessment [77]. Where a health service is publicly funded, efforts to limit non‐participation have included issuing policy directives to provide services such as MAiD when deemed in the public interest to do so and laws prohibiting non‐participating services from obstructing access [77]. These strategies seek to balance the rights and interests of health services with the rights of eligible adults to access a legal option.

The impact on HCPs who participate in MAiD extends beyond conflicts of conscience. Participating in MAiD was reported as being complex, resource‐intensive and emotionally challenging [47, 67]. Assessing the legal criteria that permit access to MAiD is one complex aspect. Eligibility criteria are generally designed to restrict access to those who are approaching the end of their life and protect members of society who may be vulnerable to abuse [79]. Crafting laws to achieve this complex objective requires language flexible enough to cover many circumstances, and yet prescriptive enough to be applied consistently. In Canada, the requirement that ‘natural death has become reasonably foreseeable’ has been interpreted by MAiD assessors and providers as relating to either the temporality or causality of death (or both), and therefore uncertain. In other cases, although the language of the law may be clear, how it applies in certain circumstances is challenging. The Dutch network of medical practitioners who are specially trained to conduct euthanasia assessments (SCEN) reported problems with meeting the due care criteria where a person has an accumulation of age‐related health problems [53]. Similarly, the MAiD laws in Australia require assessing HCPs to prognosticate life expectancy. Prognosticating can be challenging if the required time frames extend beyond when usual clinical indicators such as anorexia, confusion, dyspnoea and reduced functional status apply. It then falls to HCPs’ clinical experience and reasoning skills [67].

Additionally, HCPs are often required to assess criteria that are not health‐related and outside their expertise, such as citizenship or residency [4]. Most jurisdictions require that the requesting person is a legal resident of the relevant jurisdiction. For example, each Australian state stipulates that the patient is both an Australian citizen and is normally resident in that state [80]. Similarly, Canadian law requires that the person is eligible to receive Canadian health services [23]. Determining whether a person meets these criteria can be time‐consuming and burdensome for assessing HCPs and impact their willingness to offer these services [28, 47]. Where HCPs seek to accommodate MAiD, they frequently corral resources from other practice areas to accommodate MAiD [66]. This is most evident in rural areas, where health services are already stretched. For example, HCPs in rural Canada are often required to work around challenges posed by rurality and leverage available resources [81]. Similar challenges have been experienced in rural Western Australia, where HCPs managing large patient loads may decide that the additional workload is overly burdensome [65]. There can also be substantial direct costs to assessing HCPs providing this service, the most prominent cost being foregone income as this service is not always appropriately remunerated, which impedes the capacity building of those willing to provide MAiD [33, 48, 82].

Where studies addressed the demand for MAiD services, there was a focus on the autonomy of the dying person who chooses that option. For a person who may choose MAiD, and is potentially eligible, information about the process is critical to avoid situations where a timely assessment is delayed, decision‐making capacity becomes impaired or the trajectory of a disease overtakes the time required for assessment [42, 71]. In systems that require prospective approval for MAiD to progress, additional administrative burdens are placed on the person seeking MAiD and the assessing HCP [68]. Having to source information about MAiD independently suggests that people require high levels of health literacy [83].

Previous research has raised concerns about the impact of health literacy on access to end‐of‐life care [83]. Although greater health literacy capabilities enable a person to choreograph and participate in meaningful social relations before they die [84], the literacy burden on those seeking MAiD can be compounded if medical practitioners are reluctant to, or prohibited from, initiating conversations about this service [55, 85]. When a HCP is silent about this service, much more is required of the person, frequently requiring support from others [86]. That support can be critical for access to MAiD and the quality of the experience; however, family caregivers often require significant support from HCPs and health systems for their support to be effective [27]. Additionally, where there are centralised, multidisciplinary MAiD teams that can support a person seeking MAiD, their families and other HCPs, it is more likely that the person will be able to access the death of their choice [27]. However, it is important that autonomy and choice in MAiD do not overshadow other important values and sociocultural influences that inform constructions of a ‘good death’ [87]. For HCPs, taking time to understand the culture, beliefs and expectations of the person seeking MAiD is central to ensuring holistic end‐of‐life care, which may include MAiD as an option.

### 4.1 Implications for Practice and Policy

Where demand for MAiD is increasing, its implementation creates numerous imposts on health services, including developing and offering staff education and support, incentivising the participation of HCPs through balanced workloads, remuneration and support for their emotional well‐being [88]. For HCPs who choose to participate, navigating complex processes while providing care for those suffering intolerably can be as rewarding as it is emotionally challenging [66]. Expanding the pool of HCPs who undertake eligibility assessments for MAiD is one option to improve access [4]. Acknowledging that such an expansion would require legislative reform, innovative service models are required to accommodate the significant burden carried by those HCPs who participate and offer layers of support for the person seeking access.

MAiD navigation services, which link those seeking MAiD with a willing HCP, have been pivotal in supporting access [69]. These roles, frequently undertaken by HCPs such as nurses, social workers and others who provide spiritual care, highlight the importance of networks of support that extend what may have been contemplated by any enabling legislation [89].

Seeking to meet the diverse needs of those at the end of life, specialist palliative care has embraced a public health approach, through the development of ‘compassionate communities’ focused on social needs and relationships, over the medicalisation of death and dying [90]. The relationship between palliative care services and MAiD has often been perceived as antagonistic, although these have similarities [91]. Given the current willingness of many societies to engage in death‐positive discourse [92], aligning MAiD with compassionate communities could represent an innovative model to address some of the barriers to access identified in this review.

### 4.2 Strengths and Limitations

This review has several strengths. Using the Healthcare Access Framework [5], the factors influencing supply and demand for MAiD were highlighted. The inclusion of only studies published in English is a limitation of this review with findings from studies published in other languages excluded.

## 5 Conclusion

Taking steps to hasten a person's death using MAiD actively challenges traditional understandings of the role of healthcare. A person's ability to access MAiD as a legal end‐of‐life option is significantly influenced by healthcare services' decisions to offer and provide the service. Furthermore, despite the number of HCPs who, in principle, may support MAiD, the administrative, financial and emotional costs of participating in MAiD limit their ability to provide it. Consequently, those seeking access must have the ability to navigate the procedural complexities in an environment where it might be difficult to find a willing and supportive HCP and at a time when the suffering associated with their life‐limiting illness is intolerable. This review has identified a unique constellation of factors that can align to influence access to MAiD. The findings invite consideration of innovative and inclusive models to promote high‐quality, compassionate care at the end of life and access MAiD if desired.

## Author Contributions

**Jayne Hewitt:** conceptualisation, methodology, data collection, data analysis and manuscript writing. **Michael Wilson:** conceptualisation, methodology, data collection, data analysis and manuscript writing. **Ann Bonner:** conceptualisation, methodology, manuscript writing and review. **Melissa J. Bloomer:** conceptualisation, methodology, data analysis, manuscript writing and review.

## Conflicts of Interest

The authors declare no conflicts of interest.

## Supporting information

Supporting information.

Supporting information.

Supporting information.

Supporting information.

## Data Availability

The authors confirm that the data supporting the findings of this study are available in the article and/or its supplementary materials.
