# Supplementary material for: Factors That Influence Access to Medical Assistance in Dying Services: An Integrative Review
Source: Health Expect. 2024 Oct 17;27(5):e70058. doi: 10.1111/hex.70058 (PMC11483748; doi:10.1111/hex.70058)
Supplement: Supplementary file 1 — Supporting information. [file HEX-27-e70058-s001.docx]

| **Example Search String** | | |
| --- | --- | --- |
| **PUBMED** | MeSH Terms | Comments or search string |
| S1 | Delivery of healthcare  OR  Delivery of health care | Captures the following terms:  Access to health services  Access to care (s)  Accessibility of health services  Health services availability  Access to treatment |
| S2 | Euthanasia, Active, Voluntary |  |
| S3 | Suicide, assisted | Assisted suicides  Death, assisted  Physician-Assisted suicide  Medically assisted suicide |
| S4 | S2 OR S3 |  |
| S5 | S4 AND S1 | ((assisted suicide [MeSH Terms]) OR (euthanasia, active, voluntary[MeSH Terms])) AND ((delivery of healthcare[MeSH Terms]) OR (delivery of health care[MeSH Terms])) |
| Limiters | 1998-2024  English |  |
