# Supplementary material for: Factors That Influence Access to Medical Assistance in Dying Services: An Integrative Review
Source: Health Expect. 2024 Oct 17;27(5):e70058. doi: 10.1111/hex.70058 (PMC11483748; doi:10.1111/hex.70058)
Supplement: Supplementary file 2 — Supporting information. [file HEX-27-e70058-s002.docx]

**Supplementary Table 1.** Characteristics of studies included in the review.

| First Author, Country | Year | Study design or method of data collection | Sample  (size, participants) | Findings relevant to answering the research question |
| --- | --- | --- | --- | --- |
| Antonacci et al., Canada | 2021 | Cross-sectional survey conducted online (email). | N = 452, HCPs (nurses, physicians), administrators and volunteers. | Participants expressed dissatisfaction with the current psychological and professional support provided by their healthcare organisation and the Ministry of Health. Additional support is needed to deal with administering MAiD and managing conscientious objections. |
| Back et al., USA | 2002 | Longitudinal semi-structured interviews. | N = 35, patients and family members | Patients valued physicians' openness to discussions about MAiD, ability to describe the natural history of the illness, care options in the last days of life, and defining reasonable expectations about dying. |
| Beernaert et al., Belgium | 2017 | Cross-sectional survey. | N = 620 (patient questionnaire), 611 (physician questionnaire). | Most patients had received information about the illness course, and treatment but only a minority got information about palliative care (12%) and euthanasia (6%). |
| Bergman et al., Netherlands | 2020 | Cross-sectional survey. | N = 498 physicians (2015), 573 physicians (across 938 questionnaires in 2016 and 2017). | Support and Consultation on Euthanasia Netherlands (SCEN) physicians perceived 21.6% of cases as difficult, particularly in assessing due care criteria. Characteristics associated with difficult cases included patients staying in the hospital, main diagnosis of heart failure/cerebrovascular accident, accumulation of age-related health problems/psychiatry/dementia, and the presence of a psychiatric disorder, or psychosocial or existential problems besides the main diagnosis. |
| Boivin et al., Canada | 2019 | Focus group followed by a 3-week online forum. | N = 50 (members of the public; citizens, patients, caregivers), N = 35 (health care professionals; physicians, nurses, pharmacists, social workers, ethicists, managers). | Concerns focused on the pressure of MAiD on vulnerable patients, support of decision-making, and roles of professionals other than physicians in MAiD. Information regarding evaluating requests included delays for evaluation, addressing patients with psychological distress, losing the capacity to consent during the process, resolving conflicts between patients, families and professionals, and conscientious objection. Participants expressed a need for information beyond the medical and legal aspects of MAiD. |
| Bolt et al., Netherlands | 2016 | Quantitative analysis of data from two cohort studies and a nested retrospective study. | N = 322 (total), N = 168 (proxies of deceased cohort members), N = 154 (cohort of people with advance directives for euthanasia). | Older adults who completed an advance directive requesting euthanasia (ADE) were more likely to have a stable preference over time compared to those who did not complete an ADE. An advanced desire for euthanasia does not necessarily result in a euthanasia request; of those requests, only a fraction may be accepted. |
| Borgsteede et al., Netherlands | 2007 | Semi-structured interviews. | N = 50. GPs (20) and patients with a life expectancy of less than 6 months (30). | Forty-four per cent of terminally ill patients did not talk with General Practitioners about euthanasia, either for religious reasons or because they did not consider it. Discussions were usually about future planning - rather than immediate requests - but usually, the discussions were quite vague. General Practitioners left it to patients to initiate discussion but were aware of signals that the patient might wish to talk. |
| Bouthillier et al., Canada | 2019 | Semi-structured interviews. | N = 22. Physicians who conscientiously objected to getting involved in a patient's MAiD requests. | Most physicians who conscientiously objected did not oppose MAiD. Rather they were in favour of patients' right to MAiD but felt uncomfortable or unable to administer MAiD. The main reason for objection was due to the emotional burden or fear of psychological repercussions of participating. |
| Brown et al., Canada | 2020 | A qualitative exploratory study with semi-structured interviews. | N = 30 (5 patients, 11 family members, 14 HCPs). | All participants had concerns regarding program sustainability, care pathway ambiguity (obtaining accurate information, referrals and delivering complexities of care), lack of support for care choices, institutional conscientious objection, and post-death documentation. Patients and family members are also concerned about the lack of advance MAiD directives, independent witness requirements on MAiD request forms and consent before administration. Healthcare professionals were also uncertain about their roles and responsibilities. |
| Brown et al., Canada | 2021a | Interpretive description methodology with questionnaire and semi-structured interviews. | N = 35; 17 Physicians, 18 NPs. | Exogenous influences on non-participation include lack of resources, uncertain/evolving practice landscape, ease of referral, institutional conscientious objection/faith-based institutions; employer-imposed practice limits and practice culture for nurse practitioners, community norms and perceptions regarding participation in MAiD. |
| Brown, et al., Canada | 2021b | Interpretive description methodology with questionnaire and semi-structured interviews. | N = 35; 17 Physicians, 18 NPs. | Endogenous influences on nonparticipation included previous personal and professional experiences, comfort with death, conceptualisation of duty (lack of alignment with professional practice/tenets or obligations to the patient), preferred end-of-life care approaches (incoherence with the vision of palliative care practices), faith or spirituality beliefs. |
| Buchbinder et al., USA | 2018 | Semi-structured in-depth interviews. | N = 19. Caregivers (family members, close friends, other closely involved individuals) of terminally ill patients who pursued MAiD. | Most patients in this study who pursued MAiD did so with support from caregivers, who are deeply implicated, socially and morally, in the process. Their support takes two forms: emotional (empathy, care, love) and instrumental (practical assistance, organisational work). These overarching forms of support were further distributed between four distinct temporal phases: preparation, ingestion (of lethal substance), waiting (witnessing death), and after death. |
| Buiting et al., Netherlands | 2008 | Online cross-sectional survey. | N = 1176 physicians (clinical specialists, general practitioners, nursing-home physicians). | Nursing home-based doctors had more difficulty than General Practitioners or specialists in clarifying the request, assessing suffering, and providing information to patients. However, they had the least trouble assessing the presence or absence of reasonable alternatives, consultation, and administration. |
| Cain et al., USA | 2020 | Survey of hospital policies. | N = 270 Californian hospitals, covering 89 unique policies. | Among hospitals permitting MAiD, 38% required safeguards beyond the legal requirements. Most hospitals provided for referral to another provider if the patient’s physician did not participate. Among hospitals not permitting MAiD, nearly all allowed providers to follow patients elsewhere to choose MAiD, and most permitted a provider to refer to another provider or system. |
| Campbell & Black, USA | 2014 | Analysis of hospice policy documents. | N = 33 Washington hospices. | There are policy differences between hospices: 1) in the language used by hospices to refer to the Washington statute that reflect differences among national organisations, 2) the values that hospice programs draw on to support their policies, 3) dilemmas created by requests by patients for hospice staff to be present at a patient’s death, and 4) five primary levels of non-involvement and participation by hospice programs in requests from patients for physician-assisted death (opposition, procedural non-participation, non-participation in physician-assisted dying, non-interference, respect patient choice). |
| Campbell & Cox, USA | 2012 | Analysis of hospice policy documents. | N = 56 Oregon hospices. | Boundaries were drawn around 6 key caregiving considerations: 1) language regarding MAiD; 2) informed decision-making by patients; 3) collaboration with physicians; 4) provision of lethal medication; 5) assistance in the patient’s act of taking the medication (most hospices placed prohibitions on the organisation and staff on procuring medication as well as staff assistance with medication self-administration); and 6) staff presence at the time of medication ingestion. |
| Campbell et al., USA | 2022 | Cross-sectional survey. | N = 300 physicians of various specialties. | Most respondents were willing to refer a request for MAiD to a qualified provider. Those who had provided MAiD found it fulfilling but time-consuming; Many found it ethically challenging; a minority found it professionally risky. Barriers to participation included lack of knowledge, emotional and time stress, ethical concerns, religious concerns, lack of support, policies of employers/practice and being known as a MAiD provider. |
| Close et al., Australia | 2023 | Analysis of policy documents. | N= 15 policies from 9 entities | Institutional objections were clearly stated in policy documents, however, they provided little practical guidance that would enable patients to navigate MAiD in light of these objections. Some institutional policies were not consistent with guidance provided by centralised bodies. |
| de Boer et al., Netherlands | 2019 | Exploratory qualitative interviews. | N = 15 general practitioners. | Six categories of pressure regarding MAiD were identified: 1) emotional blackmail, 2) control and direction by others, 3) doubts about fulfilling the criteria (needing more time to form a judgement about criteria fulfilment), 4) counterpressure by patient’s relatives, 5) time pressure around referred patients (lack of time to form a trusting relationship) and 6) organisational pressure (trouble fitting euthanasia in around schedule). |
| Dees et al., Netherlands | 2013 | In-depth semi-structured interviews. | N = 90 interviews of 32 cases (31 patients, 31 relatives, 28 treating physicians). | Five key themes regarding a request for MAiD were identified: 1) initiation of sharing views and values about euthanasia and assisted suicide (most physicians reacted to patients' requests rather than initiated), 2) building relationships as part of negotiating (positive outcomes were noted when there was clear communication), 3) fulfilling legal requirements, 4) detailed work of preparing and performing MAiD and 5) aftercare and closing. |
| Dion, et al., Canada | 2019 | Semi-structured interviews and surveys. | N = 21 participants (8 MAiD assessors, 1 patient, 7 support persons, 5 MAiD administrators). | Support for persons and patients thought MAiD was acceptable via telemedicine; assessors were positive but expressed some reservations (concerns about empathy in an online consult, unable to get to know the person). Support persons and patients thought MAiD was appropriate for their situation. Assessors and administrators said it related to specific patient characteristics. All participants thought telemedicine increased access to MAiD assessments (limited physical capacity). |
| Dobscha et al., USA | 2004 | Semi-structured interviews. | N = 35 Oregon physicians. | Sources of discomfort for physicians included concerns about adequate symptom management, not wanting to abandon patients, and an incomplete understanding of patient preferences, especially when they did not know patients well. Participation required a large time investment and was emotionally intense. Support was rarely sought from colleagues, and emotional support was from physicians' spouses. |
| Fischer et al., Switzerland | 2009 | Quantitative analysis of reported assisted suicides. | N = 165 cases of assisted suicide. | The main reasons that people sought MAiD were pain, to prevent the need for long-term care, neurological symptoms, immobility, and dyspnoea. More patients than doctors said that other considerations were control of circumstances, loss of dignity, weakness, less engagement in activities that make life enjoyable, loss of concentration and insomnia. |
| Frolic et al., Canada | 2022 | Anonymous online survey and qualitative focus groups. | N = 300 HCPs (survey); N = 53 (39 HCP focus group participants, 14 family member face-to-face interviews (16 family members in total); N = 4 (interviews with 3 hospital corporation's executive leadership team, 1 board member familiar with the MAiD program). | Family and healthcare professionals felt supported by the hospital's program. Most felt that there was respect for moral diversity regarding MAiD. However, objectors noticed that they had suddenly become a 'minority' due to the rapid uptake of MAiD processes. Palliative care doctors most strongly identified as objectors at the beginning, but they increasingly accepted the process over time. A key strength of the program was the interprofessional team model for its service delivery. |
| Gamondi et al., Switzerland | 2019 | Semi-structured interviews. | N = 23 palliative care physicians. | Older doctors (>50 years) saw requests about MAiD related to information needs rather than obtaining assisted suicide; Most would not initiate the discussion. Alternatives were presented as "natural" deaths, and most would set boundaries early in the relationship. Topics of discussion between doctors and patients included: i) interaction with Right-to-Die societies, ii) being present at the death, iii) certification of condition and capacity iv) reasons for the patient's request, v) offer of alternatives, and vi) consultations with families. No physicians reported having specific training to manage assisted dying requests. |
| Ganzini et al., USA | 2009 | Anonymous survey. | N = 56 patients requesting PAD or a PAD advocacy organization. | Chief reasons for pursuing MAiD were loss of independence, wanting to control the time and manner of death and dying at home, and the prospect of worsening pain or quality of life and the inability to care for themselves. At the interview, physical symptoms (e.g., pain, dyspnoea, fatigue) were rated unimportant. |
| Gerson, et al., USA | 2019 | Semi-structured interviews are conducted face-to-face or via phone. | N = 21 mixed sample of healthcare practitioners (7 nurses, 7 social workers, 4 physicians, 3 chaplains). | Most participants found it difficult to distinguish between deaths from suicides and deaths that were intentionally hastened. In relation to access/affordability, high costs of medication prohibit some patients from accessing MAiD, difficulty obtaining a prescription (professionals placed in the unexpected position of responsibility and power over the decision to prevent suicide or identify options), unable to find a pharmacy willing to dispense prescriptions or doctor to sign paperwork, institutional limitations (policy preventing discussion of MAiD). |
| Haining et al., Australia | 2023 | Qualitative exploratory design, using semi-structured interviews. | 29 participants from four main stakeholder groups: patients and families, health practitioners, regulators, and MAiD system personnel. | Early experiences and reflections of key stakeholders suggest that while many of the regional initiatives implemented by Western Australia are largely effective in addressing regional access inequities, challenges for regional MAiD provision and access remain. |
| Khoshnood et al., Canada | 2018 | Semi-structured interviews conducted via phone. | N = 16 physicians (were present for at least 1 MAiD procedure). | Three main themes were identified. 1) Professional relationships were enhanced, except for relationships with those who conscientiously objected, which were strained; 2) Lack of compensation for time-intensive tasks (e.g. documentation, assessments, pre-briefing, debriefing); 3) workload increases leading to sacrificed personal time. The scarcity of MAiD providers in rural areas meant physicians may have to travel long distances to remote/underserviced jurisdictions to ensure patient access. Lack of billing codes for MAiD-related services. |
| Kortes-Miller., Canada | 2022 | Qualitative exploratory design, using semi-structured interviews. | 23 physicians involved in MAiD practice | The introduction of MAiD has added a layer of complexity to healthcare practice. Participating in MAiD requires substantial commitment, given the time and resources required. |
| Kusmaul, et al., USA | 2021 | Quantitative analysis of MAiD bills. | N = 23 legislative bills regarding MAiD legislation introduced to the US Congress between 1994 and 2020. | Most bills either aimed to restrict the use of federal funds, regulate the drugs commonly used for MAID, prohibit the development of policies or practices supporting MAID, or regulate practitioners’ roles in MAID. These bills either directly prevent access to MAiD by preventing implementation of MAiD or practitioners from advocating for MAiD (acceptability), legally supporting or using research to support MAiD. No bills supported the purpose of MAiD (opportunity cost). |
| Lees, et al., Canada | 2022 | Retrospective cohort study. | N = 218 patients (all patients in Nova Scotia who requested MAiD in 2018, were deceased at the time of analysis, did not withdraw their request, and were not deemed formally ineligible for the procedure). | The median number of days of survival from request to death was 8.0 days (IQR=11.5) for those with an incomplete assessment, whereas for those deemed eligible, median survival from request to determination of MAiD.  Eligibility was also 8.0 days (IQR= 16.0). Median time survival from MAiD request to death in those with a complete assessment was 23.5 days (IQR = 50.8). |
| Lees, et al., Canada | 2021 | Retrospective cohort study. | N = 383 patients (all patients in Nova Scotia who requested MAiD between June 2016 and December 2018). | Consultation with palliative care services, cohabitation, and Charlson Comorbidity Index (CCI) > 6 was associated with decreased odds of completing MAiD. |
| Lemiengre et al., Belgium | 2008 | Content analysis of policy documents. | N = 42 policy documents from Belgian (Flemish) hospitals. | All policies described their hospital’s stance on euthanasia in competent terminally ill patients though few described their stance in incompetent terminally and non-terminally ill patients. Catholic hospitals restrictively applied the euthanasia law with palliative procedures and interdisciplinary deliberations. Few policies made directions regarding psychological or spiritual support for caregivers during the euthanasia care process. |
| Munro et al., Canada | 2020 | Retrospective cohort study. | N = 84 patients (requested MAiD at The Ottawa Hospital between February 2016 and June 2017). | Patients who completed MAiD were more likely to cite physical suffering as the reason for request than those who failed to complete MAiD. The Ottawa Hospital palliative care team was involved in 46.4% of patients who requested MAiD. |
| Oczkowski et al., Canada | 2021 | Semi-structured interviews conducted online. | N = 20 mixed sample of physicians (MAiD assessors, providers, family physicians), RNs, NPs, social workers, and clinical managers. | Three themes were identified. 1) Enhancing patient access and experience through a centralised MAiD referral portal and emotional support for pts and families; 2) Supporting providers and sustainability through balanced caseloads and appropriate remuneration (as well as continuing education, feedback, observation, peer debriefing and emotional support for staff); 3) institutional supports such as comms tools and standardized care pathways (also early identification and training of new MAiD assessors and providers) |
| Oliver et al., International (Europe, North America, Australia) | 2022 | Mixed methods study consisting of a cross-sectional survey administered online and online/phone semi-structured interviews. | N = 89 (survey of a mixed sample of HCPs).    N = 18 (interviews with HCPs and agencies providing assisted dying and euthanasia services. | Service flexibility and nimbleness were key in maintaining access. Calculated ‘rule-breaking’ was considered justifiable to maintain access during COVID-19 restrictions (e.g. telemedicine was used to increase access in places even where it was illegal to do so). Most practitioners continued to provide MAiD services during the pandemic, and when they withdrew from engagement, it was due to contagion risk or to care for sick relatives. |
| Pearlman et al., USA | 2005 | Longitudinal case studies using semi-structured interviews. | N = 60 participants (patients and family members), 35 patients, and 159 interviews. | Seven common influential issues regarding motivation to undergo PAS within 3 categories were identified: illness-related experiences, changes in the person's sense of self, and fears about the future. The pursuit of PAS was motivated by multiple interactive factors in the context of progressive, serious illness. Patients considered hastened death over prolonged periods and repeatedly assessed the benefits and burdens of living vs dying. |
| Perron et al., Canada | 2023 | Mixed online-questionnaire. | N = 245 professionals involved in MAiD practice | The variability of practices between interdisciplinary support groups creates issues for access and variable quality of support available to those involved in MAiD. |
| Redelmeier et al., Canada | 2021 | A population-based case-control study. | N = 50 096 palliative care patients of whom, 920 received MAiD. | There was a 39% decreased odds of receiving MAiD associated with low socioeconomic status (OR=0.61, 95% CI 0.50 to 0.75, p<0.001), across patient groups adjusted for age, sex, home location, diagnosis, and frailty. Mean judgments of suffering were higher when assessing a rich patient than a poor patient. |
| Roest & Leget, Netherlands | 2023 | Interpretive epistemological approach using semi-structured interviews. | N = 10 health professionals who were not GPs | Decision-making around physician-assisted suicide is never straightforward. Rather, it is a complex and multifaceted process involving a range of health professionals. |
| Ruijs et al., Netherlands | 2014 | A prospective study to quantify unbearable suffering. | N = 64 patients (estimated to die within six months). | Unbearable symptoms were present in 94% of patients with an explicit request for euthanasia or assisted suicide and in 87% of patients without an explicit request. No differences were found in the prevalence of unbearable suffering for physical, psychological, social, and existential symptoms, nor for overall unbearable suffering, between patients who did or who did not explicitly request euthanasia or assisted suicide. |
| Rutherford et al., Australia | 2023 | Semi-structured interviews. | N = 25 Victorian physicians (of various specialties), with no in-principal objection to MAiD | There main themes were identified from the interviews. 1) New challenges to medical practice; 2) Multiple barriers to access ; 3) Willingness to participate is situation-specific |
| Sellars et al., Australia | 2022 | Semi-structured interviews conducted online or via phone. | N = 32 Victorian physicians involved in the MAiD process during the first 12 months since it became legal in Victoria. | Five major themes were identified. 1) a nascent approach to care; 2)practising within clinical and legal uncertainty; 3) confronting practices; 4) personal sacrifices and coping amid new challenges. A major tension was not just how doctors’ perceptions impacted their well-being and satisfaction, but also how these challenged their continued involvement in assisted dying. |
| Shaw et al., Canada | 2018 | Exploratory descriptive design using semi-structured interviews. | N = 8 physicians who offered MAiD in British Columbia in 2014. | Three main themes were identified: 1) providing MAiD is rewarding 2) the ability to provide is complicated and challenging; 3) the law should be updated. Key issues affecting access: refusal of faith-based organizations, disagreements with conscientious objections, dealing with family member/friend grief, and legislative restrictions. |
| Silvius, et al., Canada | 2019 | Comparison of policies and MAiD practices. | Not stated | Common themes between MAiD programs in different regions included 1) protection of vulnerable patients' rights, 2) family involvement, 3) confidentiality, 4) privacy, 5) conscientious objection, 6) right to practice rituals and cultural beliefs, and 7) sensitive and compassionate communication among medical practitioners, patients, and families. |
| Smith et al., USA | 2015 | Cross-sectional survey. | N = 55 Oregon patients who either requested MAiD or contacted a MAiD organisation/service.  N = 39 individuals with advanced disease who did not pursue MAiD. | Patients requesting MAiD had higher levels of depression, hopelessness, and dismissive attachment. MAiD requesters were more likely to be unmarried, have lower levels of spirituality, and be more highly educated. |
| Snelling et al., New Zealand | 2023 | Qualitative exploratory design using semi-structured individual and focus groups | N = 26 healthcare professionals | Adoption of open and transparent organisational policies, ongoing education of the workforce, and measures to reduce the stigma associated with assisted dying are necessary to facilitate high-quality services. |
| Snijdewind et al., Netherlands | 2018 | Cohort study using data from application forms and registration files from the Right to Die Netherlands clinic. | N = 645 patients requesting MAiD or euthanasia whose cases concluded during the study period. | Patients with a somatic condition or cognitive decline had the highest percentage of granted requests. In contrast, patients with a psychological condition had the smallest percentage of granted requests. One-third of requests from patients were ‘tired of living’. Within the group of patients with rejected requests, psychological suffering and loneliness were the most common types of suffering. |
| ten Cate et al., Netherlands | 2017 | Analysis of semi-structured interviews from a prior study (KOPPEL). | N = 33 physicians (general practitioners). | Dominant influences on General Practitioners' decisions about requests include 1) Perceived legal criteria (discrepancy between the law and what General Practitioners thought was the law, e.g. requirement of unbearable pain, not just psychological/existential suffering); 2) Individual interpretations of the legal criteria (e.g. carefully considered could be interpreted as repeatedly done over a longer period); 3) non-legal considerations (e.g., relationship with the patient, input from family, patient's state of mind). |
| Thomas et al., Canada | 2023 | Policy analysis. | N = 17 MAiD policies from 9 provinces and 3 territories | Policies described the healthcare professions that could be involved in MAiD, and the importance of an interdisciplinary approach. The primary role of central coordination services is to facilitate timely access to MAiD by linking applicants to assessors and providers. Policies focussed on ensuring patients could make an informed choice about MAiD, and encouraged assessors to carefully explore reasons why MAiD might be requested. |
| Trachtenberg & Manns, Canada | 2017 | Modelling study using published data from the Netherlands and Belgium and mortality data for the Canadian population. | Mortality data for the Canadian population, End of life cost data, and data from the Netherlands and Belgium. | If Canadians use MAiD to the same extent as people in the Netherlands and Belgium, the national health budget savings could be in the tens of millions of dollars. Providing MAiD in Canada should not result in any excess financial burden and should remain at least cost-neutral, if not leading to substantial savings. |
| Tran et al., Canada | 2022 | Respective cohort study. | N = 408 patients who had received a formal MAiD assessment at the LHSC between June 2016 and December 2019. | People from lower socio-economic positions were more likely to request a MAiD assessment, but that position did not impact the proportion who received or did not receive MAiD. This finding also held for age. Demand is higher among lower socio-economic groups, but socio-economic status does not impact access. |
| van den Ende et al., Netherlands | 2021 | Prospective study, with repeated measures of general well-being and health, persistence of the wish for PAD and mortality. | N = 66 patients between December 2016 and January 2020 who had their request for MAiD at the Euthanasia Expertise Center declined. | Most patients claimed a high desire for MAiD 12 months after being denied, and none at 12 months said their desire for MAiD did not exist anymore. Forty-four per cent consulted their General Practitioner again about the wish in the past 6 months and 19% another physician. Seventy-two per cent who consulted a physician indicated request would not be assessed again. |
| White et al., Australia | 2023a | Semi-structured interviews. | N = 28 interviews with 32 family caregivers and one patient about the experiences of 28 patients seeking access to MAiD | The major barriers to access were finding trained and willing doctors to assess eligibility for voluntary assisted dying; the time required for the application process; prohibition on telehealth consultations; institutional objections to voluntary assisted dying and the statutory prohibition on health practitioners initiating a conversation about voluntary assisted dying. Major facilitators were supportive care navigators; finding a supportive co-ordinating practitioner; the Statewide Pharmacy Service; |
| White et al., Australia | 2023b | Semi-structured interviews. | N = 28 interviews with 32 family caregivers and one about the experiences of 28 patients seeking access to MAiD | Participants reported institutional objections affecting eligibility assessments, medication access, and completing MAiD. Objections resulted in delays, transfers, and choices between progressing a MAiD application and receiving palliative or other care. |
| White et al., Australia | 2021 | Semi-structured interviews conducted online. | N = 32 physicians (of various specialties) involved in providing lawful assisted dying during the first year of the Victorian MAiD system. | The prospective approval system created several barriers to access (e.g., bureaucratic, bad online system design, caused unnecessary delays, not apt for very ill persons - length of time too demanding for patients), but it did protect doctors' safety. |
| Wiebe et al., Canada | 2021 | Semi-structured interviews. | N = 16 healthcare professionals (14 physicians, 1 NP, 1 MAiD coordinator) who provided or coordinated MAiD before and during the COVID-19 pandemic. | Requestors suffered from isolation and reduced services during the pandemic; providers were frustrated by the distancing requirements at a time when closeness was required. Some adaptations persisted with telemedicine and virtual witnessing. There were increased challenges for providers to establish rapport during the pandemic when using telemedicine for assessments and personal protective equipment for in-person care. |
