# Supplementary material for: Factors That Influence Access to Medical Assistance in Dying Services: An Integrative Review
Source: Health Expect. 2024 Oct 17;27(5):e70058. doi: 10.1111/hex.70058 (PMC11483748; doi:10.1111/hex.70058)
Supplement: Supplementary file 3 — Supporting information. [file HEX-27-e70058-s003.docx]

**Supplementary Table 2**. Quality appraisal scores

| Quality Assessment Scores* | | | | | | | | | | | |
| --- | --- | --- | --- | --- | --- | --- | --- | --- | --- | --- | --- |
|  |  | Reviewer scores | |  |  | Reviewer scores | |  |  | Reviewer scores | |
| **Design** | **First Author** | **1** | **2** | **Design** | **First Author** | **1** | **2** | **Design** | **First Author** | **1** | **2** |
| **Quantitative** | Antonacci R | 6 | 3 | **Qualitative** | Back AL | 11 | 11 | **Mixed-Method** | Brown J | 11 | 11 |
|  | Beernaert K | 7 | 7 |  | Boivin A | 7 | 7 |  | Brown J | 10 | 10 |
|  | Bergman TD | 10 | 10 |  | Borgsteede SD | 8 | 6 |  | Campbell CS (2014) | 11 | 11 |
|  | Bolt, EE | 11 | 9 |  | Bouthillier M-E | 7 | 7 |  | Campbell CS (2012) | 6 | 7 |
|  | Buiting H | 5 | 5 |  | Brown J | 8 | 6 |  | Dion S | 11 | 11 |
|  | Cain C | 6 | 5 |  | Buchbinder M | 10 | 10 |  | Frolic A | 11 | 11 |
|  | Campbell, EG | 9 | 11 |  | Close E | 11 | 11 |  | Lemiengre J | 9 | 9 |
|  | Fisher, S | 7 | 7 |  | Dees MK | 11 | 10 |  | Oliver P | 12 | 12 |
|  | Ganzini, L | 8 | 8 |  | de Boer ME | 11 | 11 |  | Silvius JL | 3 | 4 |
|  | Lees C | 6 | 6 |  | Dobscha SK | 11 | 11 |  |  |  |  |
|  | Lees C | 10 | 10 |  | Gamondi C | 10 | 10 |  |  |  |  |
|  | Munro, C | 9 | 9 |  | Gerson S M | 9 | 9 |  |  |  |  |
|  | Perron C | 10 | 9 |  | Haining CM | 10 | 11 |  |  |  |  |
|  | Redelmeier DA | 11 | 11 |  | Khoshnood N | 11 | 11 |  |  |  |  |
|  | Ruijs CD | 11 | 11 |  | Kortes-Miller,K | 11 | 10 |  |  |  |  |
|  | Smith KA | 9 | 9 |  | Kusmaul N | 11 | 10 |  |  |  |  |
|  | Snijdewind, MC | 11 | 11 |  | Oczkowski S J | 11 | 11 |  |  |  |  |
|  | Trachtenberg AJ | 8 | 7 |  | Pearlman R | 11 | 11 |  |  |  |  |
|  | Tran M | 11 | 11 |  | Roest B | 11 | 10 |  |  |  |  |
|  | van den Ende C | 9 | 9 |  | Rutherford J | 11 | 11 |  |  |  |  |
|  |  |  |  |  | Sellars M | 11 | 11 |  |  |  |  |
|  |  |  |  |  | Shaw J | 11 | 11 |  | * A score of one was allocated if the criterion was met. Maximum score = 11 Caldwell et al (2011) | | |
|  |  |  |  |  | Snelling J | 10 | 11 |  |  |  |  |
|  |  |  |  |  | ten Cate K | 11 | 11 |  |  |  |  |
|  |  |  |  |  | Thomas R | 11 | 11 |  |  |  |  |
|  |  |  |  |  | White BP (a) | 11 | 11 |  |  |  |  |
|  |  |  |  |  | White BP (b) | 11 | 11 |  |  |  |  |
|  |  |  |  |  | White BP (c) | 11 | 11 |  |  |  |  |
|  |  |  |  |  | Wiebe E | 10 | 10 |  |  |  |  |
