# Supplementary material for: Factors That Influence Access to Medical Assistance in Dying Services: An Integrative Review
Source: Health Expect. 2024 Oct 17;27(5):e70058. doi: 10.1111/hex.70058 (PMC11483748; doi:10.1111/hex.70058)
Supplement: Supplementary file 4 — Supporting information. [file HEX-27-e70058-s004.docx]

**Supplementary Table 3.** Supply and demand dimensions influencing access to MAiD.

| **Dimension** | **No. of Studies** | **Representative findings** |
| --- | --- | --- |
| **Supply Dimensions** | | |
| Approachability  *Transparency, outreach, information, screening* | 32 | Significant challenges were noted in obtaining accurate MAiD information, referrals, and delivering the complexities of MAiD care. This was compounded by an ‘air of secrecy,’ HCP lack of knowledge regarding care processes and occasionally HCP nonparticipation (Brown et al., 2020 p.1470).  Some participants described that the regional setting was conducive to MAiD provision. The nature of such communities meant that regional health practitioners often have a pre-existing therapeutic relationship with the patient and professional relationships with local health staff, which assist the process to operate more smoothly (Haining et al., 2023 p.5 of 9). |
| Acceptability  *Professional values, norms, culture, gender.* | 30 | Physicians often commented on the unusual and often unsettling experience of conversing with patients who were alert and functional and asking for a hastened death, as opposed to responding to requests for termination of life support from more imminently terminal patients or families of patients (Dobscha et al, 2004 p.454). |
| Availability and accommodation  *Geography, accommodation, hours of opening, appointment mechanisms* | 18 | The ambiguity of not knowing *if or how* MAiD fit within their practice influenced their prioritization of MAiD continuing education and their overall participation perspectives (Brown et al., 2021 p.2281).  Knowing that I’m the only one in [town] who can provide this…I was taking a holiday for a month, and I was distressed on both his and my behalf that perhaps I wouldn’t be available when he needed this done. (Koretes-Miller et al., 2022 p.e166). |
| Affordability  *Direct costs, indirect costs, opportunity costs* | 13 | Using telemedicine eliminated stressful travel costs, time and discomfort for both patients and assessors (Oliver et al., 2022 p.13).  Participants indicated that they had provided AD “pro bono”, provided AD within an existing clinical role…and one doctor reported privately billing patients for AD (Sellars et al., 2022 p.6). |
| Appropriateness  *Technical and interpersonal quality, adequacy, coordination, and continuity.* | 25 | ‘‘Our support team (social worker/chaplain) was not offered any specialized training on how to deal with the residual effects of MAiD on the remaining family members’ (grief support)’ (Antonacci et al., 2019 p.154).  Overwhelmingly doctors described the Victorian prospective oversight and approval process as bureaucratic – a ‘byzantine bureaucratic process’ – or ‘highly legalistic’ (White et al., 2021 p.9). |

| **Demand Dimensions** | | |
| --- | --- | --- |
| Ability to perceive.  *Health literacy, health beliefs, trust, and expectations* | 14 | Cohort members with an advance directive for euthanasia were more likely to request euthanasia if they were unable to accept things as they were; had the feeling of being a burden to others…were specifically worried about loss of dignity, pain, or dyspnea (Bolt et al., 2016 p.1630). |
| Ability to seek.  *Personal and social values, culture, gender, autonomy* | 14 | 96% of participants discussed their attitudes about PAD with family and friends, and 86% indicated that the people closest to them would probably support their choice to pursue PAD (Ganzini et al., 2009 p.490).  Patients who completed MAiD were more likely to live at home with family than home alone or in a nursing home (Munro et al., 2020 p.839). |
| Ability to reach.  *Living environments, transport, mobility, and social support.* | 10 | There was no significant difference between those who completed the [MAiD] procedure and those who experienced a natural death in terms of age, sex, access to a primary care provider, underlying condition, area of residency…or specialist palliative care involvement (Lees et al., 2022 p.246).  Telemedicine facilitated access to MAiD assessments for their loved ones because of their limited physical capacity, ‘I couldn’t have gotten him in the car, gotten him onto the ferry (Dion et al., 2019 p.E724). |
| Ability to pay.  *Income, assets, social capital, health insurance.* | 5 | Nurses, physicians, and social workers spoke about disparities in health care, including the high cost that often prohibits patients from pursuing MAiD ‘…so this medication that was used early on was $280 when the Death with Dignity law was passed. It is now $3000’ (Gerson et al., 2020 p.682).  We found that most MAiD assessment requests at London Health Sciences Centre (LHSC) came from patients classified into the lower SES category; however, overall, most patients who requested MAiD did not end up receiving it (Tran et al., 2022 p.632). |
| Ability to engage.  *Empowerment, information, adherence, caregiver support.* | 13 | Social support provided by caregivers on the day of an AID death fell into two broad categories: emotional and instrumental. Emotional support included expressions of empathy, care, and physical presence. Instrumental support entailed practical assistance and organizational work undertaken to facilitate the patient’s wishes and preferences for death (Buchbinder et al., 2018 p.939).  When physicians, patients, and relatives established effective relationships, positive relational effects resulted, even if initial requests were declined. Effective relationships included mutual respect for autonomy, clear communication, and collaboration (Dees et al., 2012 p.31). |
